# Case report: Sintilimab combined with anlotinib as neoadjuvant chemotherapy for metastatic bone tumor resection in patients with PSC

**DOI:** 10.3389/fimmu.2024.1372279

**Published:** 2024-05-02

**Authors:** Zheming Bao, Xiuchun Yu, Kai Zheng, Kai Zhai, Haocheng Cui, Ming Xu

**Affiliations:** Orthopedics Department, 960th Hospital of People's Liberation Army (PLA) Joint Service Support Force, Jinan, China

**Keywords:** neoadjuvant chemotherapy, non-small-cell lung cancer, pulmonary sarcomatoid carcinoma, programmed death-ligand 1, tyrosine kinase inhibitor, sintilimab, anlotinib

## Abstract

**Background:**

Pulmonary sarcomatoid carcinoma (PSC) is a rare subtype of non-small-cell lung cancer (NSCLC), which is resistant to chemotherapy and radiotherapy with a poor prognosis. PSC is highly malignant and is prone to recurrence even after surgery. The programmed death-ligand 1 (PD-L1) tumor cell proportion score (TPS) 5%, TERT and TP53 gene mutations were detected in this patient accompanied by multiple metastatic sites. The anlotinib is a novel multitarget tyrosine kinase inhibitor (TKI) that could be effective for advanced NSCLC and some sarcoma patients. Limited clinical trials and case reports have shown that PSC patients with gene mutations and PD-L1 expression have good responses to multitarget antiangiogenic drug and immune checkpoint inhibitors (ICIs). In this article, we reported a case with metastatic PSC diagnosed by Computed Tomography (CT)-guided needle biopsy treated with immunotherapy combined with antiangiogenic drugs as a neoadjuvant chemotherapy (NACT). PSC is controlled and the patient achieves successfully limb salvage treatment by surgical resection. Therefore, targeted therapy and immunotherapy can provide sufficient surgical opportunities for limb salvage in the treatment of metastatic PSC patients.

**Case summary:**

A 69-year-old male diagnosed with malignant bone tumor in the proximal femur was admitted to our hospital in June 2022 with recurrent fever as well as swelling and pain in the left thigh for twenty days. The initial computed tomography (CT) scan of the chest showed a pulmonary cavity (20 mm × 30 mm) and scattered lung masses. Subsequently, he underwent a CT-guided needle biopsy to distinguish the essence of osteolytic bone destruction and soft tissue mass in the left proximal femur which showed metastatic sarcomatoid carcinoma histology. Genetic testing revealed TERT c.-124C mutation (abundance 8.81%), TP53 p.R342 mutation (abundance 11.35%), tumor mutational burden (TMB) 7.09 muts/Mb, microsatellite stability (MSS), and PD-L1 (SP263) TPS 5% were also detected. The patient was tentatively treated with a combination of antiangiogenic drug and PD-1 inhibitor. After one course, the tumor volume significantly reduced in magnetic resonance imaging (MRI) and pathological fracture occurred in the femur after combined treatment. The patient received proximal femoral tumor resection and prosthesis replacement after defervescence. Sequentially sintilimab with anlotinib were administered for over 1 year. Finally, the local tumor was well controlled, and no obvious drug-related adverse reactions were observed. The lesions in the lung remained in partial response (PR) for more than 16 months and complete response (CR) of metastatic tumor in the proximal femur was observed through imaging examinations.

**Conclusion:**

This is the first reported case of a metastatic PSC in femur showing a favorable response to the treatment consisting of anlotinib combined with sintilimab. This case suggests that antiangiogenic therapy combined with immunotherapy may benefit patients with metastatic PSC in the preoperative adjuvant therapy for limb salvage.

## Introduction

PSC is a rare, highly malignant tumor which accounts for approximately 0.1%–0.5% of lung malignant cancers (1). PSC exhibits features of both epithelial and mesenchymal tumors and is categorized as a subtype of poorly differentiated NSCLC contains sarcoma-like elements including pleomorphic carcinoma, spindle cell carcinoma, giant cell carcinoma, carcinosarcoma, and pulmonary blastoma (2). The principles of the treatment for metastatic sarcomatoid lung cancer are the same as for other metastatic carcinomas. Surgery is the first choice of treatment when clinically appropriate. Conventional treatments and chemotherapy have limited efficacy in PSC, leading to a poorer prognosis than other subtypes of NSCLC. Meanwhile, reports of the potential benefit of immunotherapy and molecular targeted therapy in PSC are very rare.

Anlotinib, as an antiangiogenic inhibitor, is a novel multitarget TKI targeting the vascular endothelial growth factor receptor (VEGFR), which is effective in the palliative treatment of advanced NSCLC and some sarcomas (3). It has been shown that vascular invasion is a major factor in the poor prognosis of PSC patients (4). PD-L1 expression is associated with improved tumor response rates and prolonged progression free survival (PFS) after immunotherapy, which may still be effective even with PD-L1 levels <1% (5). Currently, the expression of PD-L1 in malignant tumors indicates a potential response to ICIs. Sintilimab is a human-derived immunoglobulin G4 (IgG4) monoclonal PD-1 antibody that blocks the binding of PD-1 to receptor (3, 6). In lung cancer patients carrying TP53 mutations, the expression of PD-L1 is significantly increased, leading to enhanced T-cell infiltration and immune sensitivity of the tumor. Therefore, patients with PD-L1 expression combined with TP53 mutations may benefit from immunotherapy. In this case, we treated metastatic PSC with the combination of anlotinib plus sintilimab which rapidly reduced the tumor volume. Although there have been anecdotal reports on the use of ICIs and TKIs in the treatment of PSC, data from clinical studies of the combination of anlotinib and sintilimab in patients with PSC are still lacking. Further, little is known about whether the combination of targeted therapy and immunotherapy can provide limb-salvage conditions for patients with metastatic tumors. We reported the dramatic response of systemic multiple metastatic PSC with comprehensive treatments including anlotinib combined with sintilimab, an antiangiogenic drug with PD-1 inhibitor. This rare case may provide for individualized treatment of patients with advanced PSC. In particular, the efficacy of immunological and antiangiogenic therapies in the preoperative treatment of limb salvage in patients with metastatic tumors can be demonstrated.

## Case presentation

### Chief complaints

A 69-year-old male patient, who was previously healthy without a history of other chronic, infectious or genetic diseases, was admitted to the hospital in June 2022 because of swelling and pain in the left thigh for twenty days, accompanied by recurrent fever. There was no cough, expectoration, wheezing or other abnormal pulmonary symptoms on admission.

### Laboratory examinations

Liver function, renal function and infectious disease screenings revealed no significant abnormalities. The white blood cells, erythrocyte sedimentation rate (ESR), procalcitonin (PCT), C-reactive protein (CRP) and other inflammatory makers were abnormally elevated. To further identify the foci of infection, we performed metagenomic detection of pathogenic microorganisms including bacteria, funguses, viruses, parasites, mycoplasmas, chlamydia and mycobacterium tuberculosis. Unfortunately, no significant abnormal pathogen nucleic acid sequences were detected. To provide further treatment to this patient, genetic sequencing testing for 551 target genes was performed. The results revealed that foci showed TERT c.-124C mutation (abundance 8.81%), TP53 p.R342 mutation (abundance 11.35%), TMB 7.09 muts/Mb, MSS, and PD-L1 (SP263) TPS 5% were detected.

### Personal and family history

The patient had a smoking history of 20 cigarettes/day for more than 30 years. No information for family history.

### Imaging examinations

**Figure 1:**
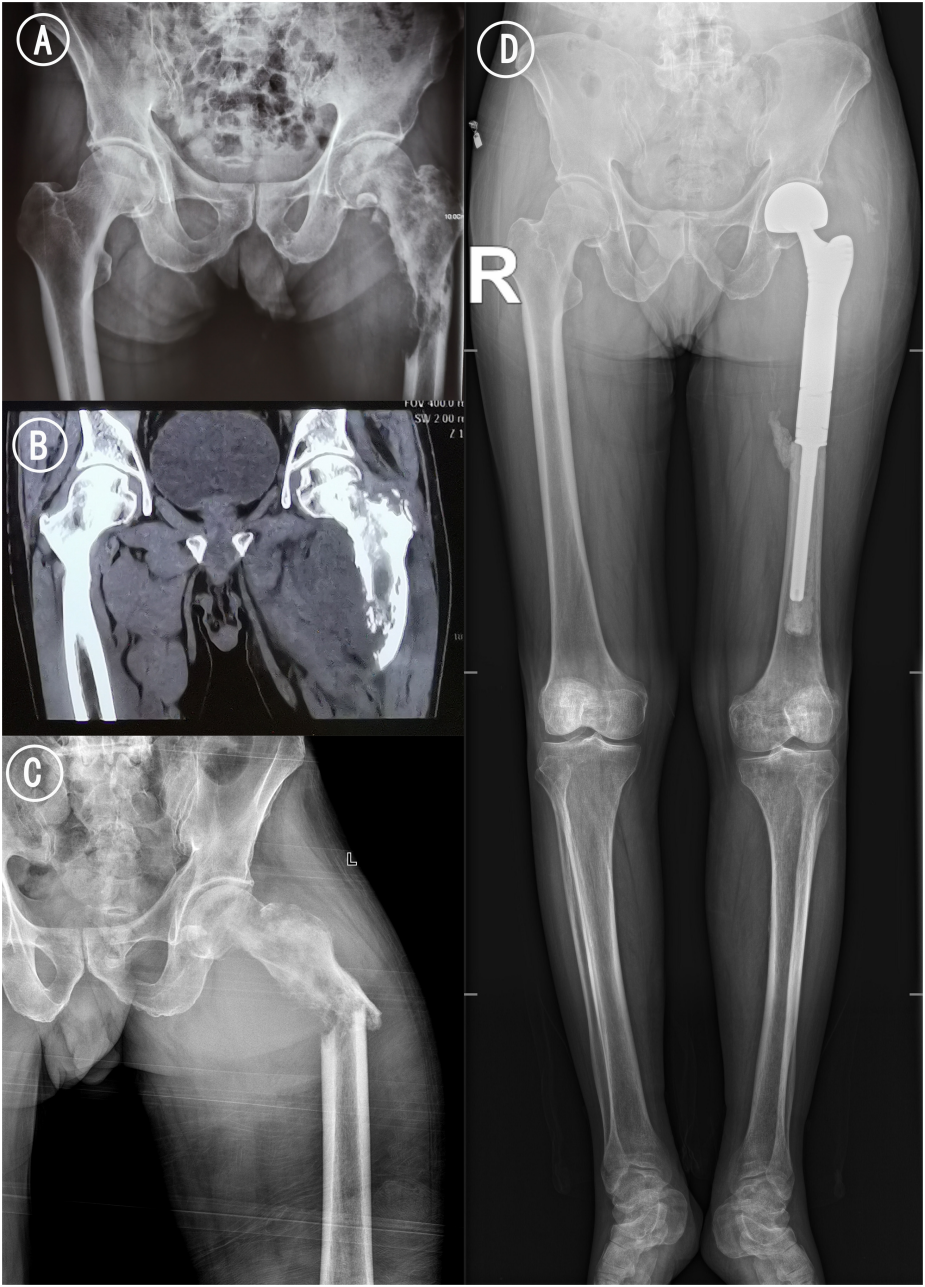
Radiograph and CT of femur. **(A)** Pelvic ap before pathological fracture. **(B)** Coronal CT scan of bilateral hip. Osteolytic bone destruction was observed in the upper left femur. **(C)** Left hip ap x-ray examination after pathological fracture. The femoral cortex was discontinuous, angulated and deformed, and the soft tissue was swollen. **(D)** Postoperative X-ray of the long-leg radiographs 15 months after treatments: the femoral prosthesis was well in place without loosening and abnormal radiolucent lines.

**Figure 2:**
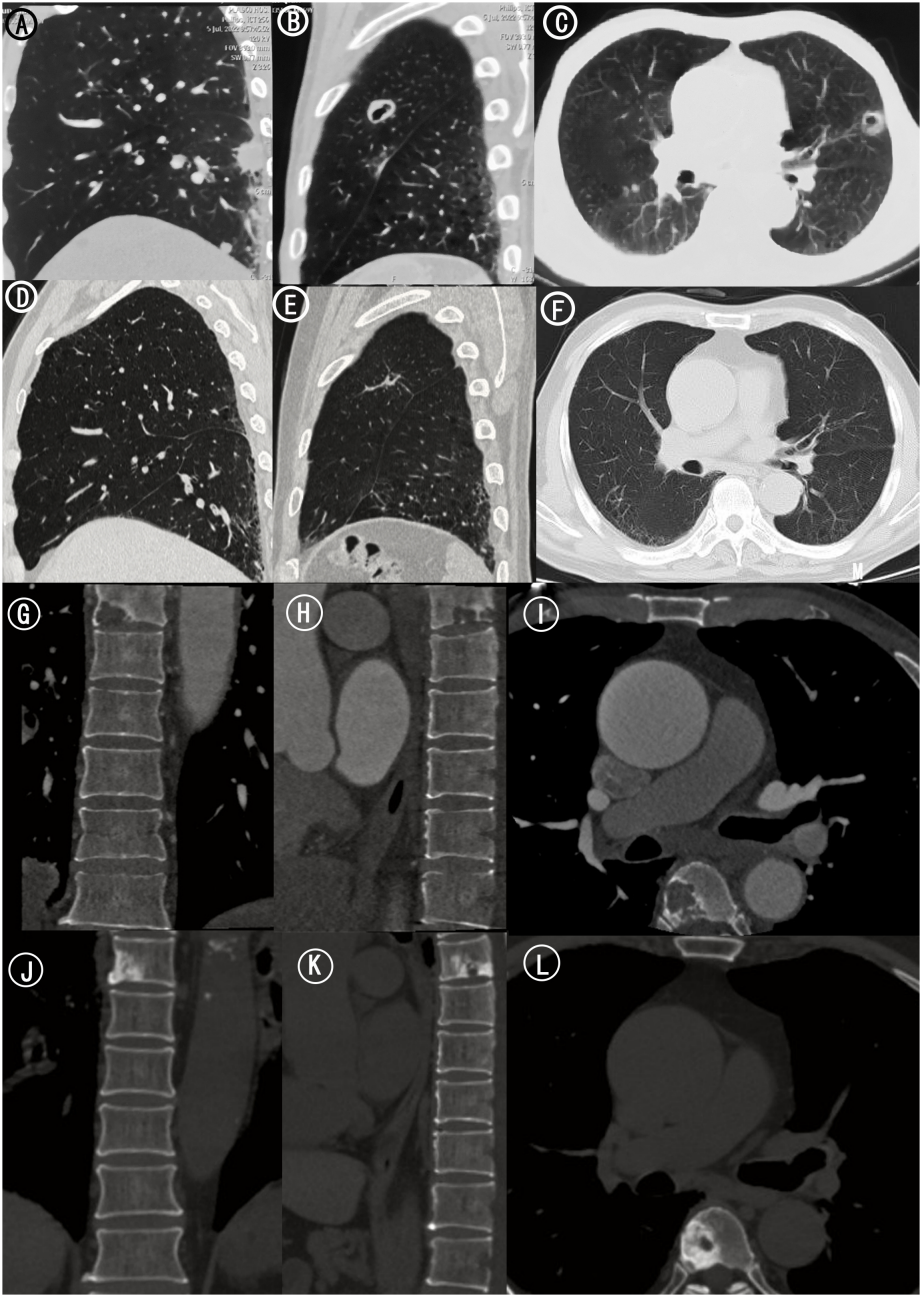
Chest and Thoracic CT. **(A)** Sagittal CT scan of left lung. Scattered lung nodules were seen. **(B)** Sagittal CT scan of left lung. **(C)** Axial CT scan of bilateral lungs. A pulmonary cavity (20 mm × 30 mm) in the superior lobe of left lung. **(D)** Sagittal CT scan of left lung 15 months after treatments. The scattered lung nodules were significantly smaller than before. **(E)** Sagittal CT scan of left lung 15 months after treatments. **(F)** Axial CT scan of bilateral lungs 15 months after treatments. The left lung cavity was further reduced in size of approximately 16*0.9mm. **(G)** Coronal CT scan of thoracic vertebra. **(H)** Sagittal CT scan of thoracic vertebra. **(I)** Axial CT scan of thoracic vertebra. Bone destruction in thoracic six vertebra. **(J)** Coronal CT scan of thoracic vertebra 15 months after treatments. **(K)** Sagittal CT scan of thoracic vertebra 15 months after treatments. **(L)** Axial CT scan of thoracic vertebra 15 months after treatments. There were signs of a circular hyperdense shadow on the right side of thoracic six vertebra.

**Figure 3:**
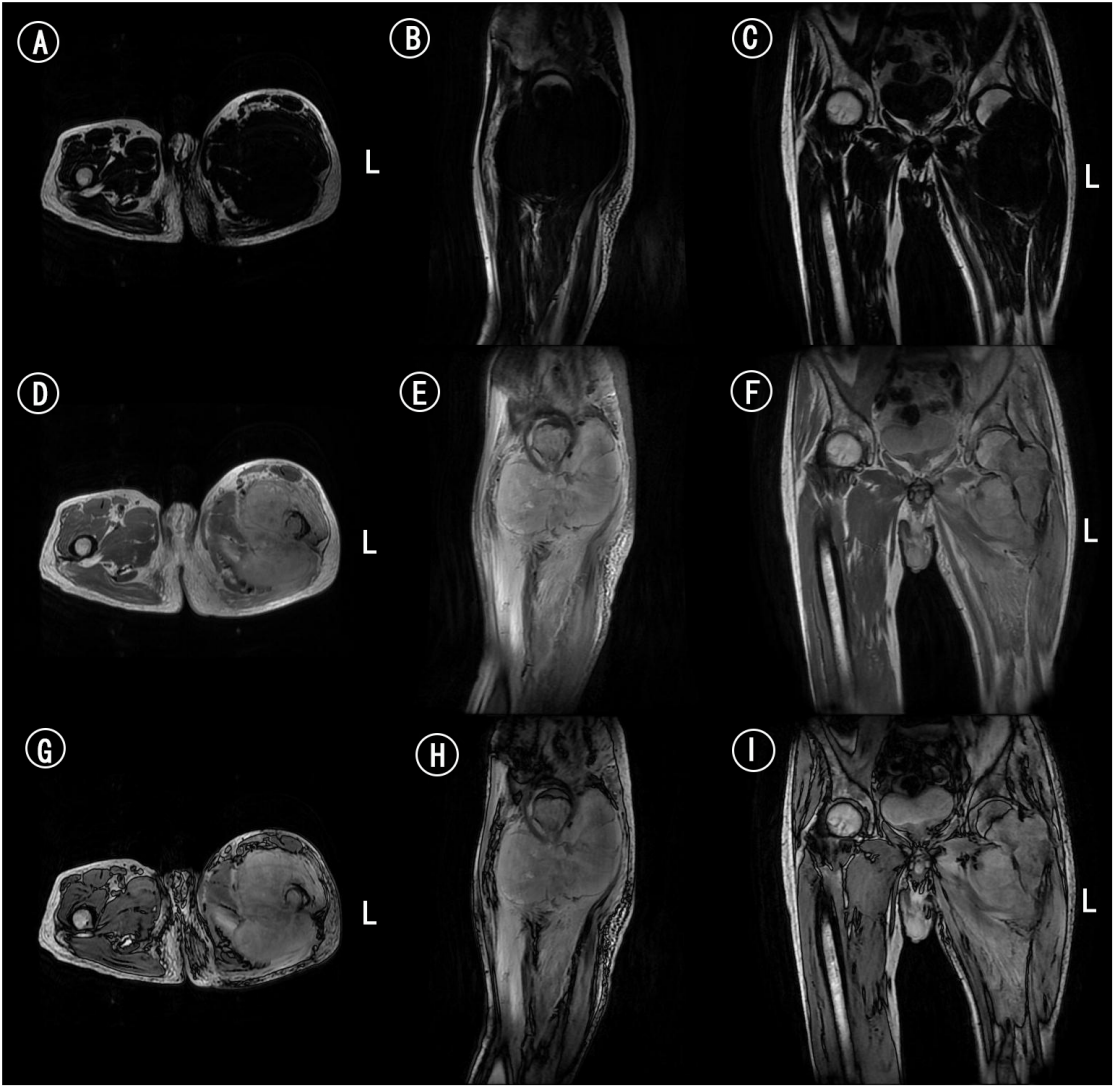
MRI of femur. **(A)** Axial MRI scan of femur in T1WI. **(B)** Sagittal MRI scan of femur in T1WI. **(C)** Coronal MRI scan of femur in T1WI. **(D)** Axial MRI scan of femur in T2WI. **(E)** Sagittal MRI scan of femur in T2WI. **(F)** Coronal MRI scan of femur in T2WI. **(G)** Axial MRI enhanced scan of femur. **(H)** Sagittal MRI enhanced scan of femur. **(I)** Coronal MRI enhanced scan of femur.

**Figure 4:**
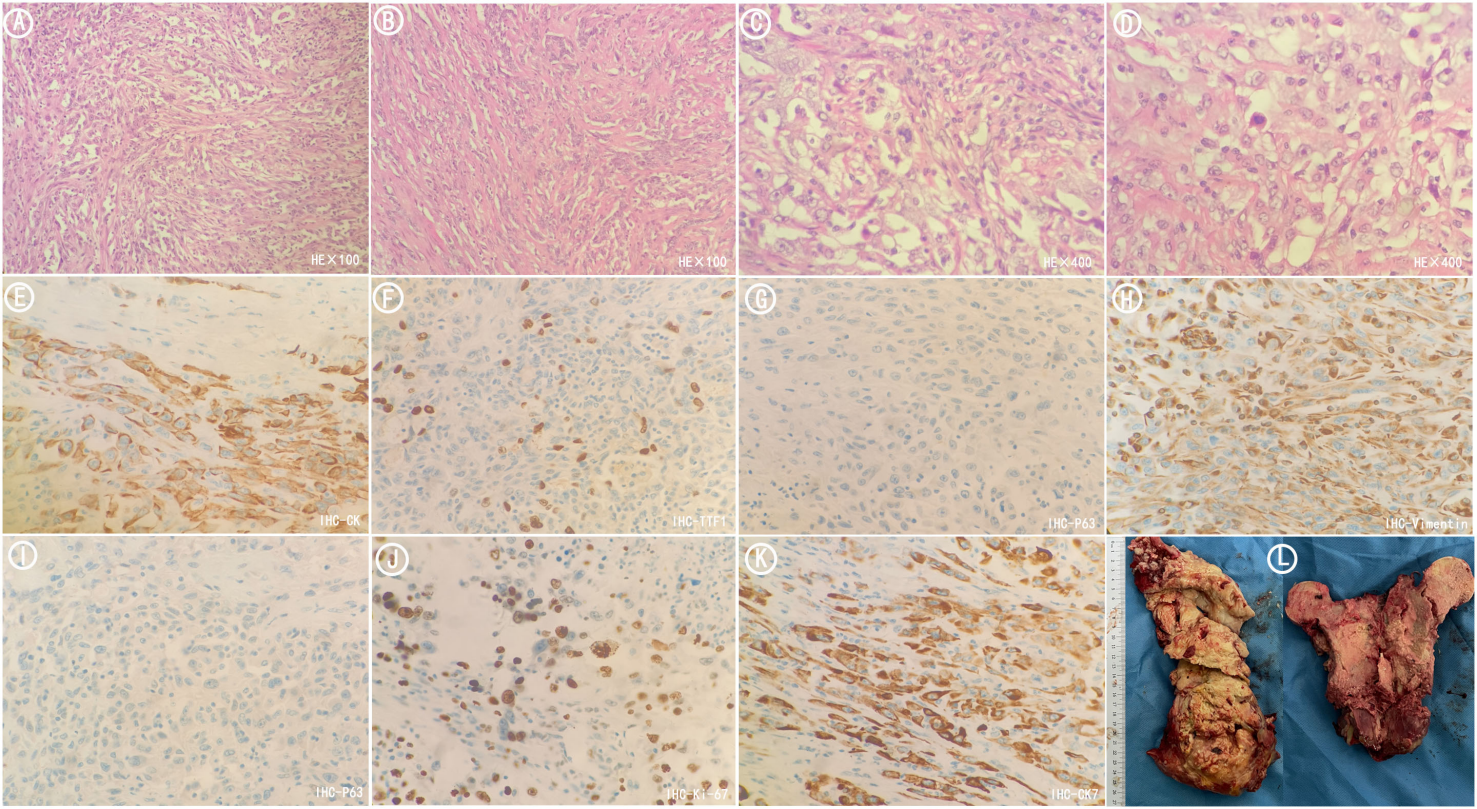
Postoperative pathological sections of metastatic tumors in femur. **(A)** HE stained sections(×100). The tumors were mainly composed of spindle-shaped cells arranged in fascicular and storiform patterns. **(B)** HE stained sections(×100). The tumors were arranged in nests and cords. **(C)** HE stained sections(×400). The tumors were arranged in nests and cords. **(D)** HE stained sections(×400). The nuclei were of variable size and vacuolated with visible nucleoli. **(E)** Immunohistochemistry CK (+). **(F)** Immunohistochemistry TTF1(focal +). **(G)** Immunohistochemistry P63(-). **(H)** Immunohistochemistry Vimentin (+). **(I)** Immunohistochemistry P40(-). **(J)** Immunohistochemistry Ki67(+). **(K)** Immunohistochemistry CK7(+). **(L)** Tumor specimens resected during surgery.

On June 27, 2022, the patient underwent computed tomography (CT) of pelvis and abdomen which revealed a bone destruction in thoracic six vertebra and left proximal femur (**Figures 1**, **2**). Further evaluation of the lung using enhanced CT scan showed a pulmonary cavity (20 mm × 30 mm) in the superior lobe of left lung and scattered lung nodules, which was considered a possible malignancy with vascular invasion multiple metastatic sites (**Figure 2**). Femoral bone metastases were also found using emission computed tomography (ECT) and magnetic resonance imaging (MRI) (**Figure 3**). The multisystem neoplastic lesion was considered as multiple metastatic foci. No obvious abnormalities were observed in head MRI. Positron emission tomography (PET)- CT showed multiple pulmonary nodules in both lungs with increased fluorodeoxyglucose metabolism. The left proximal femur and thoracic six vertebra were hypermetabolic and metastasis to these sites was considered. The CT-guided percutaneous needle biopsy of the femur was performed on July 8, 2022. Microscopically, it showed the tumors were composed of spindle cells and epithelioid cells. Immunohistochemical staining showed TTF1(focal +), CK (+), CK8/18(+), Vimentin (+), CK7(+), EMA (+), TLE1(+), CD56(-), CD34(-), NapsinA (-), Desmin (-), P63(-), P40(-), S100(-), Ki67(+, about 50%) (**Figure 4**). The features were consistent with PSC.

### Diagnosis

The patient was diagnosed with PSC (T4N3M1c, stage IVB) with metastasis to left proximal femur and thoracic six vertebra.

### Treatment

Due to the unresectable locally femoral neoplasm, we then decided to begin preoperative NACT. On July 30, the patient began taking anlotinib (Focus V®, Jiangsu Chia-Tai Tianqing Pharmaceutical and Advenchen Laboratories) 8 mg peros (po) once daily continuously for 2 weeks of each 3-week cycle (q3w) combined with immunotherapy consisting of sintilimab (Tyvyt®, Innovent Biologics and EliLilly and Company) 200 mg once every 21 days.

Surprisingly, after one course treatment with sintilimab combined with anlotinib, pathologic fracture of the left proximal femur occurred at the site of tumor invasion (**Figure 1**), and the symptom of fever of undetermined origin was relieved. There were signs of osteosclerosis at the margins of the vertebral destruction. In order to relieve the pain of the fracture and restore mobility, an urgent resection of the femoral tumor and prosthesis replacement for proximal femoral were performed. Postoperative pathology sections and immunohistochemistry confirmed that the tumor in the proximal femur was metastatic sarcomatoid carcinoma (**Figure 4**).

### Outcome and follow-up

Since then, the regimen of anlotinib 12 mg orally quaque die(qd) from day 1 to 14 combined with sintilimab 200 mg on day 1 every 3 weeks has been maintained. The latest postoperative follow up to our hospital was on December 27, 2023(15 months after treatments), during which the chest-abdomen-pelvis CT showed that the left lung cavity was further reduced in size of approximately 16*0.9mm with scattered foci of fibrosis and focal inflammation in both lungs. The scattered lung nodules were also significantly smaller than before (**Figure 2**). The range of vertebral body destruction which showed a circular hyperdense shadow on the right side of thoracic six vertebra was not significantly changed (**Figure 2**). No signs of new metastasis and significant abnormalities were found in the original proximal femoral tumor site and other vertebral bodies.

Currently, the cancer has been stable for over 15 months and no notable discomfort occurred while receiving sintilimab combined with anlotinib.

## Discussion

Antiangiogenic agents combined with PD-1 inhibitors have shown synergistic antitumor effects and have been proposed as an effective strategy for the treatment of NSCLC (7–10). However, the efficacy of this combination in neoadjuvant chemotherapy before tumor resection has not been reported. PSC is a rare and poorly differentiated NSCLC, which is commonly accompanied by sarcoma or sarcomatoid components, characterized by rapid proliferation, high vascular invasion and epithelial-mesenchymal transition. Many previous case reports have shown that compared with surgical resection or combined chemotherapy, combination therapy with TKIs and ICIs have promising efficacy in patients with PSC, significantly improving the progression free survival (PFS) and overall survival (OS) of patients, and some even achieve PR (8, 11).

The efficacy of immunotherapy is closely correlated to the expression of PD-L1. Immune checkpoint proteins which are involved in immune regulation of tumor, can promote antitumor immunity. Among them, PD-1 may be utilized by tumor cells expressing PD-L1 to evade immune surveillance (3). ICIs can improve the prognosis of patients with advanced NSCLC. The high expression of PD-L1 and proportion of patients with high TMB are relatively common in PSC compared with other subtypes (8). It has been reported that among PSC patients, over 50% showed positive PD-L1 expression (12). Therefore, we proposed that PSC is expected to benefit from immunotherapy.

Sintilimab is a novel highly selective humanized IgG4 monoclonal antibody that blocks the interaction between PD-1 and its ligand by binding site of programmed cell death PD-1, thereby restoring the function of endogenous T cell and eliminating tumor cells (3, 6). It has been proven to have encouraging antitumor activity and clinically beneficial in the treatment of advanced solid tumors including advanced NSCLC (13, 14).

As is consistent with data reported in other studies, the most common mutation in PSC is the TP53 gene (7, 15). The next generation sequencing (NGS) results for the patient in this study revealed the TP53 gene mutation (abundance 11.35%), which is associated with benefit from immunotherapy. We measured PD-L1 expression to determine the sensitivity of tumor cells to immunotherapy. In our patient, the PD-L1 TPS≥1% in metastatic femoral neoplasm sections indicated that the patient was likely to achieve favorable response to sintilimab.

Anlotinib is a novel TKI with a broad-spectrum of targeted inhibitory effect on cell growth and angiogenesis such as vascular endothelial growth factor receptor (VEGFR) and fibroblasts growth factor receptor (FGFR) (3). VEGF not only promotes angiogenesis but also inhibits T cell differentiation and dendritic cell maturation, reduces tumor T cell infiltration and regulates immune response by increasing immune suppressor cells. It helps tumor cells evade immune surveillance and ultimately leads to an immunosuppressive tumor microenvironment (3, 16). As a novel multitarget antiangiogenic drug, anlotinib is currently approved as a third-line treatment of NSCLC by the National Medical Products Administration (NMPA) (3, 14). It can down-regulate the expression of PD-L1 on vascular endothelial cells, reducing hypoxia, increasing CD8+ T cell infiltration, inhibiting recruitment of tumor-associated macrophages and enhancing the immune surveillance on tumor cells, thereby inhibiting the tumor (14).

A growing amount of evidence describes the significantly synergistic therapeutic benefits against a variety of tumors between sintilimab and anlotinib (3, 11, 14, 17). Antiangiogenic agents plus PD-1 inhibitors showed promising activity in patients with advanced soft tissue sarcoma (STS) (18). Accordingly, we speculated the potential by which the synergistic effect of anlotinib and sintilimab plays an antitumor role in the neoadjuvant chemotherapy of metastatic sarcomatoid carcinoma.

In this case, CT-guided percutaneous femoral biopsy was performed. Spindle cells and epithelioid cells were shown microscopically. Epithelial-related markers TTF-1 (focal+), CK (+), CK8/18(+), CK7(+), EMA (+), and the mesenchymal-related marker Vimentin (+), CD34(-), Desmin (-) were shown immunohistochemically. Therefore, the final pathological report diagnosed metastatic sarcomatoid carcinoma (spindle cell carcinoma).

Sintilimab has been approved by the NMPA for patients with locally advanced or metastatic nonsquamous and squamous NSCLC. Therefore, we attempted to administrated sintilimab combined with anlotinib treatment, a PD-1 inhibitor with antiangiogenic drug, and favorable results were obtained. After one cycle of treatment, the body temperature returned to normal, the thigh circumference was significantly reduced, and the pain symptoms were relieved. Unfortunately, pathological fracture occurred. We believe that the occurrence of pathological fracture was caused by shrinkage and necrosis in the tumor during immunotherapy and targeted therapy, rather than tumor proliferation. Tumor necrosis was found in the femoral neoplasms resected during the operation, and no tumor tissue was found at the resection margin. These proved that the NACT of anlotinib and sintilimab combination therapy was effective. The efficacy of combination therapy was also confirmed by follow-up for over 1 year. There was no peripheral recurrence in the left thigh, the pulmonary cavity was reduced, and the pulmonary nodules partially disappeared after 1 year of treatment, indicating the ability of ICIs and TKIs combination therapy.

To our knowledge, this is the first case showing a favorable response to sintilimab combined with anlotinib which was used as neoadjuvant chemotherapy for metastatic bone tumor resection. In general, the combination of sintilimab plus anlotinib has exhibited encouraging efficacy, durability, and tolerability in patients with advanced NSCLC. Immunotherapy combined with antiangiogenic drug may be a promising strategy and an effective way as NACT for treating patients with PSC.

## Patient perspective

During the early phase of my disease, I experienced recurrent fever and pain in the left thigh, which was not controlled by multiple antibiotics. However, during the treatment with the combination of sintilimab and anlotinib, my fever relieved. After completing limb salvage surgery, I was able to engage in activities of daily living including performing household chores, walk up and down stairs on my own without the use of a walker. At present, my symptoms related to the disease are well-controlled and no pulmonary symptoms such as cough occur. During the regular combination therapy, I have no nausea and vomiting.

## Data availability statement

The original contributions presented in the study are included in the article/supplementary material. Further inquiries can be directed to the corresponding author.

## Ethics statement

The studies involving humans were approved by Ethics Committee of 960th Hospital of PLA Joint Service Support Force. The studies were conducted in accordance with the local legislation and institutional requirements. The participants provided their written informed consent to participate in this study. Written informed consent was obtained from the individual(s) for the publication of any potentially identifiable images or data included in this article.

## Author contributions

ZB: Writing – original draft, Writing – review & editing. XY: Writing – original draft, Writing – review & editing. KZhe: Writing – original draft. KZha: Writing – original draft, Writing – review & editing. HC: Writing – original draft. MX: Writing – original draft, Writing – review & editing.
